# Analysis of the Nosema Cells Identification for Microscopic Images

**DOI:** 10.3390/s21093068

**Published:** 2021-04-28

**Authors:** Soumaya Dghim, Carlos M. Travieso-González, Radim Burget

**Affiliations:** 1 Signals and Communications Department (DSC), Institute for Technological Development and Innovation in Communications (IDeTIC), University of Las Palmas de Gran Canaria (ULPGC), Las Palmas de Gran Canaria, 35001 Canary Islands, Spain; soumaya.dghim101@alu.ulpgc.es; 2 Department of Telecommunications, Faculty of Electrical Engineering and Communication, Brno University of Technology (BUT), 61600 Brno, Czech Republic; burgetrm@vutbr.cz

**Keywords:** image processing, Nosema disease, machine learning, deep learning, image, disease detection

## Abstract

The use of image processing tools, machine learning, and deep learning approaches has become very useful and robust in recent years. This paper introduces the detection of the Nosema disease, which is considered to be one of the most economically significant diseases today. This work shows a solution for recognizing and identifying Nosema cells between the other existing objects in the microscopic image. Two main strategies are examined. The first strategy uses image processing tools to extract the most valuable information and features from the dataset of microscopic images. Then, machine learning methods are applied, such as a neural network (ANN) and support vector machine (SVM) for detecting and classifying the Nosema disease cells. The second strategy explores deep learning and transfers learning. Several approaches were examined, including a convolutional neural network (CNN) classifier and several methods of transfer learning (AlexNet, VGG-16 and VGG-19), which were fine-tuned and applied to the object sub-images in order to identify the Nosema images from the other object images. The best accuracy was reached by the VGG-16 pre-trained neural network with 96.25%.

## 1. Introduction

Several deadly diseases endanger honeybees. Possibly one of the best known is Nosema. Nosema, which is also called Nosemiasis or Nosemosi [1], is caused by two species of microsporidia, *Nosema apis* (*N. apis*) and Nosema ceraena (*N. ceraena*) [2]. Several works were published regarding the impact of Nosema disease on commerce, society and food, as shown in [3,4], and the disease is currently of one the major economic importance worldwide [5]. The health of the two species of bees is a particular interest of biologists, not only because of their significant role in the economy and food production but also because of the vital role they give in the pollination of agricultural and horticultural crops. Many biological descriptions of its DNA and its behavior can be found in literature, for example in [6,7]. Furthermore, several recent works try to treat this disease using a chemical simulation, as presented in [8,9].

Furthermore, from a computer science point of view, honeybees are of significant interest. Several works were, for example, involved in bees and controlling their behavior [10]. The study presented monitoring the behavior of bees to help people associated with beekeeping to manage their honey colonies and discover the bee disturbance caused by a pathogen, Colony Collapse Disorder (CCD) or colony health assessment. In [11], many tools of image analysis were explored to study the honeybee auto grooming behavior. Chemical and gas sensors were used for measurement. Destructor infestations are applied inside the honeybee colony to detect disease. The study was based on measurements of the atmosphere of six beehives using six types of solid-state gas sensors during a 12-h experiment [12]. Regarding the image processing of Nosema disease part, there are currently two major works. In [13], the authors used the Scale Invariant Feature Transform to extract features from cell images. It is a technique that transforms image data into scale-invariant coordinates relative to local features. A segmentation technique and a support vector machine algorithm were then applied to microscopic processed images to automatically classify *N. apis* and *N. ceranae* microsporidia. In [14], the authors used the image processing techniques to extract the most valuable features from Nosema microscopic images and apply an Artificial Neural Network (ANN) for the recognition, which was statistically evaluated using the cross-validation technique. The last two works used image processing tools for feature extraction and Support Vector Machine (SVM) and ANN for classification. Today the traditional tools of machine learning like ANN, Convolutional Neural Network (CNN), and SVM are frequently used in human disease detection [15], especially in medical image classification of Heart diseases [16], Alzheimer disease [17] and Thorax diseases [18]. Deep learning approaches were used in [19] for semantic images segmentation. This work used the Atrous convolutional Neural Network for segmentation and some pre-trained NN for validation like PASCAL-Context, PASCAL-Person-Part and CityscapesDeep. In [20], a method using a 2D overlapping ellipse was implemented using the tools of image processing and applied to the problem of segmenting potentially overlapping cells in fluorescence microscopy images. Deep learning is an end-to-end machine learning process that trains feature extraction together with the classification itself. Instead of organizing statistics to run through predefined equations, deep learning uses multiple layers of processing data and setting fundamental parameters on knowledge records, and it trains the computer to analyze and recognize data. Deep learning approaches are widely applied in the analysis of microscopic images in many fields: human microbiota [21], material sciences [22], microorganism detection [23], cellular image processing [24] and many other important works in this field. Deep learning techniques have accelerated with transfer learning the ability to recognize and classify several diseases. The objective of this paper is to validate this hypothesis. 

All the methods of Nosema detection and recognition presented by the biologists in the literature were either molecular detections or genetic descriptions. This paper evaluates two different strategies for automatic identification of the Nosema cell disease based on the microscopic images. First, images of Nosema cells and the existing objects have been cropped from the principal microscopic images. Using these images, the first dataset has been built. Then, the obtained images were processed again and several different features have been extracted. These features were used to create a second dataset. The obtained databases were used for the evaluation recognition of the Nosema cells. The first approach uses a model, which uses the extracted features by an ANN and an SVM. The second approach uses the deep learning and transfer learning methods: first, CNN, and then pre-trained networks AlexNet, VGG-16 and VGG-19. The tools of transfer learning used by authors reached notable results as this is the first time they have been used for the purpose of Nosema cell recognition.

The main innovation of this paper is the evaluation of two different strategies of automatic detection and recognition Nosema cells from microscopic images and identification of the robust and successful approach as a robust methodology for automated identifying and recognizing Nosema cells versus the other existing objects in the same microscopic images. 

The rest of the paper is organized as follow: Section 2 describes the dataset preparation. In Section 3 is described dataset, segmentation, features extraction, ANN training, the use of SVM, CNN, the use of Alex Net, VGG-16 and VGG-19. The experiments are described in Section 4. Section 5 discusses the obtained results. Finally, the paper is concluded.

## 2. Materials: Preparation of The Dataset

**Figure 1:**
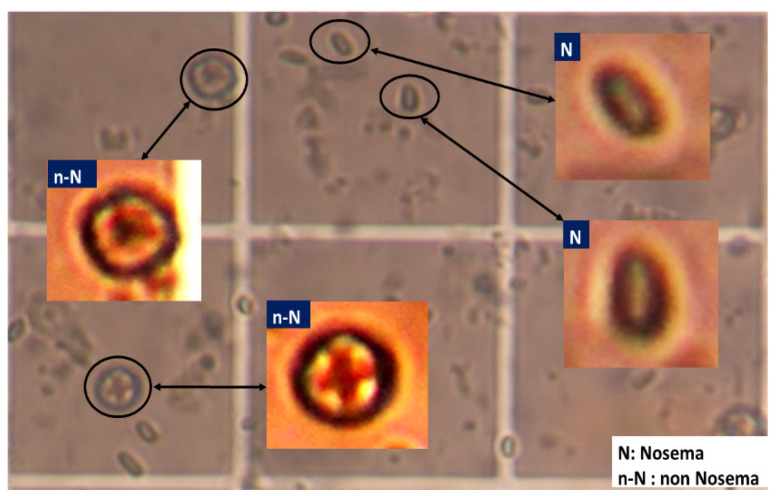
Example of extraction of Nosema cells and other existing objects in a part of one microscopic image.

For the experiment, Nosema microscopic images were used. So far, it is not known whether these images contain a sufficient amount of information for accurate detection and recognition of the disease cells. It was only known that the important information was diffused all over the image and behind the majority of unimportant data. The used images in this work are 400 RGB images, encoded with JPEG and with a resolution of 2272 × 1704 pixels. Each sample was labelled by one of the 7 classes, according to the severity of the disease or the number of disease cells present in the microscopic image. From these 400 RGB images, a set of sub-images have been extracted. To do that, each microscopic image was divided into many smaller images forming subdivisions of the existing and clear objects. This first phase was done manually due to the low quality of input images by cropping the object of interest (i.e., cells). All the existing objects in the microscopic images were extracted as sub-images and labelled whether they stand for: Nosema(N) and not Nosema cells (n-N), see Figure 1. The area chosen was as small as possible, where an isolated and clear microscopic cell is located. Then, in the second automatic phase, the selected objects are processed to prepare them for the segmentation process (see Figure 1).

Based on the steps described above, a dataset containing 2000 sample images in total was created. It consists of 1000 Nosema cells samples and 1000 images, which are not Nosema cells, i.e., any other existing objects in the microscopic images. Table 1 below shows information about the extracted sub-images for dataset construction.

The microscopic sub-images were examined using two strategies:

- The first strategy is based on an image processing approach, where features were extracted manually.
- The second set of strategies is based on the use of the whole sub-image and the deep learning.

**Figure 2:**
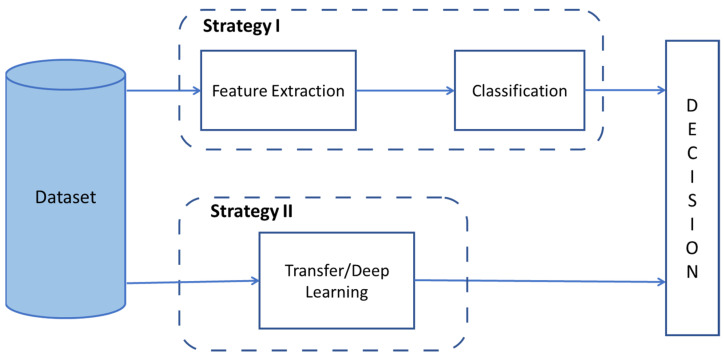
Implemented Strategies for Nosema Recognition.

Figure 2 shows strategies covered in the paper.

## 3. Methods

In the scope of this study, two different strategies were implemented. All the methods are shown according to both of the strategies. The methods are working on the dataset of sub-images (2000 images). 

### 3.1. Strategy 1: Nosema Cells Recognition with Image Processing and Machine Learning

This subsection is divided into two parts. The first part describes how the features were extracted and prepared for the training of a model. The second part shows the proposed classification systems. 

#### 3.1.1. Preprocessing for Feature Extraction

**Figure 3:**
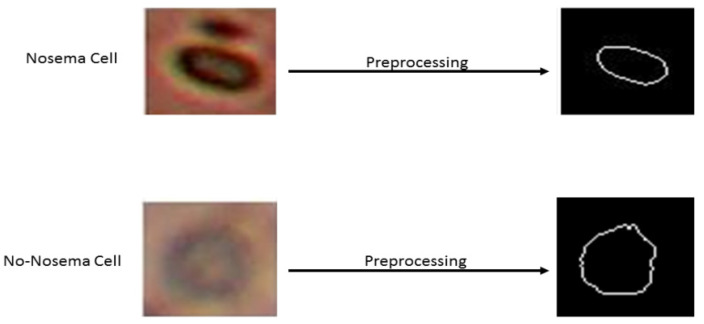
Shape results of two examples before and after preprocessing. The first sample is Nosema and the second is non Nosema object.

A preprocessing stage is necessary before extraction of the features. The initial point is an RGB image. The first step is to convert the image from RGB to a grayscale image. The second step consists of binarization of the image by the thresholding using the Otsu method [25]. In the third step, the flood-fill operation was used on background pixels of the input binary image to fill the object hole from its specific locations and then to ignore all smaller existing objects in the image of the desired object. As the final step, the object perimeter is enhanced using the dilatation method [26]. So, the desired shape of the object is obtained by calculating the difference between the two images, before and after perimeter enhancement. The result of the final step is a shape image, which was extracted from the sub-image of the dataset (see Figure 3).

From the shape image, in total 9 features were extracted. They describe the structure of the Nosema cell and consist of 6 geometric and 3 statistic features. Furthermore, from the extracted sub-images, 6 texture features and 4 Gray Level Co-occurrence Matrices (GLCM) color features were calculated.

##### Geometric Features Extraction

The geometric features describe basic characteristics of geometric form. They are also the most significant for us because, after several experiments, the best results were achieved using them. These parameters were used and defined in [14] respectively:

- The size/the perimeter: given that the shape of the Nosema cell is similar to an ellipse form and the other objects have different rounds shapes, perimeter formula of an ellipse adopted have been adopted in this study. This calculation is based on *a* and *b* variables where *a* is the semi-major axis and *b* is the semi-minor axis. Perimeter *P* is given by the following equation:
  (1) $$P=\;\pi \cdot \sqrt{2\cdot {\left(a^{2}+b\right)}^{2}\;}$$
- Area A is given by the following formula:
  (2) A= π·a·b 
- Relation R is the dividing quotient of the height (H) and width (W) of the shape.
  (3) R= H/W 
- The equivalent diameter (D), which is the diameter of the circle with the same area of the object,
  (4) $$D=\sqrt{4\times \frac{A}{\pi }}\;$$
- The solidity (S): it is the portion of the area of the convex region contained in the object,
  (5) $$S=\frac{A}{\mathrm{convex}\;\mathrm{area}}$$
- The eccentricity (E): it is the relation between the distance of the focus of the ellipse and the length of the principal axis. Let $f=1-\frac{a}{b}$ in which *a* is the semi-major axis and *b* is the semi-minor axis of the ellipse.
  (6) $$E=\sqrt{f\times \left(2-f\right)}$$

##### Statistic Features Extraction

The remaining features 7, 8 and 9 were calculated using the polar coordinates of the object, in particular, the polar coordinates of a Cartesian point (x, y). Let us say that a point *M* is at such a distance (*r*) and such a direction (*θ*) of the point of origin (*o*) of the reference point. It is a projection or a one-dimensional representation of the boundary. This is found by computing the distances from the centroid (center of “mass”) of the object to the boundary as a function of angles in any chosen increment. The resulting set of distances, when properly scaled, was the vector needed as distances of the angle to the boundary pixel. 

After that, a value for these distances is truncated, which are the nearest integers to a value to calculate the last three respective parameters.

- The standard deviation of these distances have been calculated and which is the feature number 7, the standard deviation is a measure of variability, or what the range of values is, it normalizes the elements of *N* along the first array dimension whose size does not equal to 1; where *P* can be a vector or a matrix and in this case is a vector of the radius values of polar coordinates of the studied object, and *E* is its mean. It is given by Equation (7):
  (7) $$Std.deviation\;\left(\sigma \right)=\sqrt{\frac{1}{N}\cdot \overset{N}{\underset{j=1}{\sum }}{\left(P_{ij}-E_{i}\right)}^{2}}$$
- The Variance $\sigma^{2}$ is the mean of the squared distances between a value and the mean of those values: it normalizes Y by *n* − 1 if *n* > 1, where *n* is the sample size or pixels shape number. This is an unbiased estimator of the variance of the population from which *x* is drawn, as long as *x* consists of independent, distributed distances. For *n* = 1, Y is normalized by *n* with *μ* is the average of all *x* values. In this case, the variance is calculated as the normalized distances between the centroid and every single pixel in the object shape.
  (8) $$\sigma^{2}=\frac{\left(x_{1}-\mu \right){\;}^{2}+{\left(x_{2}-\mu \right)}^{2}+{\left(x_{3}-\mu \right)}^{2}+\ldots +{\left(x_{n}-\mu \right)}^{2}}{n}$$
- The Variance derivate is the derivate that calculates the difference and the approximate derivative of the variance (X), for a vector X, is [X(2) − X(1) X(3) − X(2) … X(n) − X(n−1)]. It is given by the following equation:
  (9) $$f’(\sigma^{2})=-n^{-2}\left[\left(x_{1}-\mu \right){\;}^{2}+{\left(x_{2}-\mu \right)}^{2}+{\left(x_{3}-\mu \right)}^{2}+\ldots +{\left(x_{n}-\mu \right)}^{2}\right]$$

##### Features Extraction: Texture and GLCM 

**Figure 4:**
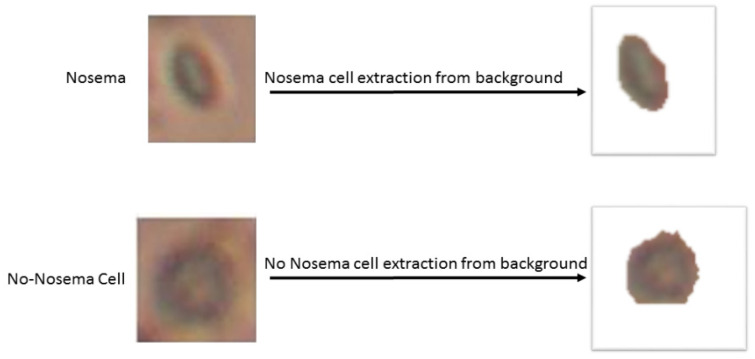
Example of a Nosema cell and non-Nosema object extraction from its backgrounds.

The next step consists of the use of the RGB object image to extract more information about texture and color. Nevertheless, before that, it is needed to separate the object from its background in the image; to do that: individual Hue (V), saturation (S) and Value (V) channels have been extracted after converting the image from RGB to HSV color spice image, then authors look for the vivid color by thresholding the V mask, after that, authors set the H and S masks to 0 and the V mask to 1 and concatenate the three new HSV channels. Finally, the authors convert back the image to RGB color image to have the object without it’s background, as shown in Figure 4:

The number of texture parameters is 6 and they are the measurement of the entropy of RGB and HSV channels; it can be defined as a logarithmic measurement of the number of states with a significant probability of being occupied. The input intensity images are the blue, red, green and yellow channels. Furthermore, the Hue and saturation masks’ randomness is calculated. The value/lightness channel was dropped since it does not give any extra information. Suppose *x_i_* is the set of pixels with the color/channel *i* of the image and *p(x_i_)* is its probability. The 6 entropy parameters are calculated by the same equation 10 above:

(10) $$E\left(x_{i}\right)=\overset{N}{\underset{i=1}{\sum }}P\left(x_{i}\right)\cdot log_{2}\left(p\left(x_{i}\right)\right).$$

As mentioned before, the Nosema cells look to be more yellow inside, that is the way a Grey Level Co-occurrence Matrix was applied to the yellow mask to extract more texture information about this color. The GLCM is very widely used as a statistical method of extracting a textural feature from images. It was used in several works of feature extraction, like in features skin extraction [27] or plant disease feature extraction [28]. GLCM is widely used to extract useful information from medical images, that is why GLCM is developed to overcome the limitations of the available extracted features and to be more accurate as indicated in [29], a novel strategy to compute the GLCM called HaraliCU can offload the computations into the Graphics Processing Units (GPU) cores, thus allowing to drastically reduce the running time required by the execution on Central Processing Units (CPUs). In [30], a developed method called CHASM exploits the HaraliCU method mentioned previously, a GPU-enabled approach, capable of overcoming the issues of existing tools by effectively computing the feature maps for high-resolution images with their full dynamics of grayscale levels, and CUDA-SOM, a GPU-based implementation of the SOMs for the identification of clusters of pixels in the image. The general rule in the statistical texture calculator says that these are calculated from the statistical distribution of combinations of intensities observed at specified positions relative to each other in the image. Based on the number of pixels in each combination, statistics are categorized into first-order, second-order, and higher-order statistics. The GLCM is a method of extracting the second-order statistical texture characteristics. Third-order and higher-order textures are theoretically possible but not commonly implemented due to computation time demands and difficulty to interpret them [31]. The GLCM is considered a greyscale image I defined in Z. The grey level co-occurrence matrix is defined to be a square matrix G_d_ of size N where, N is the total number of grey levels in the image. The (*i*, *j*) th entry of G_d_ represents the number of times a pixel X with intensity value *i* is separated from a pixel Y with intensity value *j* at a particular distance k in a particular direction d. Where the distance k is a non-negative integer and the direction d is specified by d = (d_1_, d_2_, d_3_, … d_n_), where d_*i*_ ∈ {0, k, −k} ∀*i* = 1, 2, 3, …, n [32]. Four features were extracted from the Haralick GLCM applied to the image of the yellow channel: contrast, correlation, energy, and homogeneity, the most significant features given by the GLCM.

(11) $$Contrast=\overset{Ng-1}{\underset{n=0}{\sum }}n^{2}\cdot \left[\overset{Ng}{\underset{i=1}{\sum }}\overset{Ng}{\underset{j=1}{\sum }}p\left(i,j\right)\right]$$

Correlation measures the linear dependency of grey levels of neighboring pixels:

(12) $$Correlation=\frac{1}{\left(\sigma i.\sigma j\right)}\cdot \underset{i}{\sum }\underset{j}{\sum }\left(i-\mu i\right)\cdot \left(j-\mu j\right)\cdot P_{i,j}\;.$$

It is also called Angular Second Moment (ASM), and it is of high value when two neighbor pixels are very similar:

(13) $$Energy=\overset{Ng-1}{\underset{i=0}{\sum }}\overset{Ng-1}{\underset{j=0}{\sum }}p{\left(i,j\right)}^{2}$$

Homogeneity is high when a local grey level is uniform:

(14) $$Homogeneity=\underset{i}{\sum }\underset{j}{\sum }P\left(i,j\right)\cdot \frac{1}{1+{\left(i-j\right)}^{2}}\;.$$

##### Segmentation Diagram Block and Recognition

The automatic approach of this part of work is to study the existing objects in the microscopic images of Nosema disease; to study both Nosema cells and other types of cells present in microscopic images, the desired objects are detected, useful features are extracted (geometric, texture and statistic features) by an automatic segmentation method, and the result is a vector of 19 features. Then, a multilayer Neural Network system is used as a classifier, the set of features in order to recognize the Nosema disease cells vs. the other objects in the images.

Once the features of the different object were extracted, the feature dataset is generated: it consists of 19 features for 2000 objects, i.e., a 38,000 value divided equally between two kind of objects: one for the calculated features of the objects of interest (Nosema cells), and the other for other existed object in the microscopic images. This part of the work was significantly computationally demanding since the extraction of 2000 sub-images as well as the calculation of 19 features for each image cost many days of computations, using a CPU, in particular, PcCom Basic Elite Pro Intel Core i7-9700/8GB/240SSD.

In this part of the paper, neural networks were used for the automatic detection of Nosema diseases in honeybees. The neural networks proved their quality in many real-world applications as well as for classification tasks. Usually, a neural network is made up of two parts which constitute the set of learning functionalities used to train the NN model, while a set of testing functionality is used to verify the correctness of the trained NN model. The appropriate network design should be configured, including network type, learning method and with one or two hidden layers. In the learning phase, the connection weights were always updated until they reached the defined iteration number or the acceptable error. Therefore, the ability of the ANN model to respond accurately was ensured by using the mean squared error (MSE) criterion to emphasize the validity of the model between input and network output. Furthermore, the network calculates the outputs and automatically adjusts the weights to reduce errors and recognize the objects.

For the experiment, the dataset was divided into a learning part of the model and another part for testing and validation. During the proposed approach, two types of experiments were conducted: in the first one, the model was tested with only the 15 geometric, statistic and texture features without counting the yellow color features calculated with the GLCM. The second experiment was implemented by concatenating all the 19 features. Furthermore, these two experiments were done to prove the strong presence of yellow color in the cell of Nosema. The experiments were done by applying different precision of the data division between data for training and the data for testing. The experiment was conducted with several different neural network architectures—in particular, it has experimented with the number of neurons in the hidden layer. Each test was repeated at least 30 times to obtain the optimal value of success recognition accuracy. First of all, the program was tested with a number of neurons equal to the number of input features extracted from the images (15 or 19) in which the weight is added randomly, and after that, the number of neurons was increased in the hidden layer by 50 in every new experiment (see Table 2).

#### 3.1.2. The Use of Support Vector Machine: SVM

Support vector machines SVM is a supervised learning algorithm used for classification and regression problems [33]. To ensure that SVM will give the optimal result, the parameters of the classifier were optimized. The optimized options have been the cost “*C*”, also called error term or regularization parameter and the kernel trick function, which calculates the dot product of two vectors in the space of very large characteristics. Different kernel functions can be specified for the decision function and the radial basis function (RBF) is commonly used, especially for nonlinear hyperplanes. RBF kernel for the SVM has been chosen, which is in the following form:

(15) $$K\left(X_{1},X_{2}\right)=exponent\left(-\gamma \cdot {\left||X_{1-}X_{2}|\right|}^{2}\right)$$

where $\left||X_{1-}X_{2}|\right|$ is the Euclidean distance between X_1_ and X_2_, and γ: gamma is used only for RBF kernel. The non-regularization of the values of “γ” and “C” will cause overfitting or an underfitting of the model. The SVM has been configured with C = 3 and γ = 5 × 10^−5^ as the architecture with the best result. In this case, the SVM model will classify two classes corresponding to Nosema cells and non-Nosema cells (or other objects).

**Figure 5:**
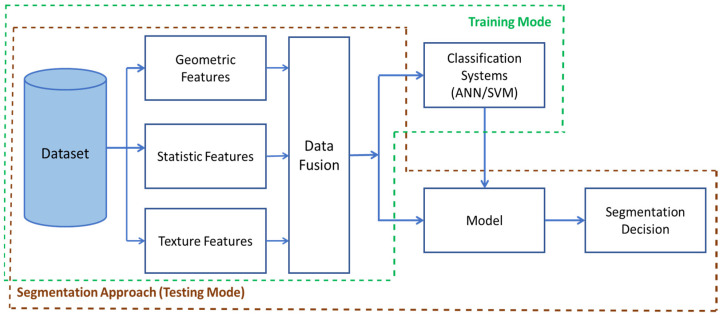
The Segmentation Diagram Block of the first strategy in Nosema detection: The Training Mode consists of the part of dataset construction, features extraction, and their fusion to be trained with ANN and SVM. The Testing Mode consists of data preparation for testing the model and decision making.

Figure 5 shows the diagram block of the processing model for ANN and SVM classification systems for the first implemented strategy.

### 3.2. Strategy 2: Nosema Cells Recognition Using Deep Learning Approaches

#### 3.2.1. Nosema Recognition with the Implemented CNN

A convolutional neural network CNN is a network architecture for deep learning which learns directly from data. They are used to classify images or to predict continuous data. In the scope of this paper, a new CNN network was designed, but before entering them into the network, input data and the predictors have been normalized were normalized. Furthermore, batch normalization layers should be used to normalize the outputs of each convolutional and fully connected layer. The architecture of a CNN should contain input layers that define the size and type of input data, the middle layers which contain the main layers of learning and computation, and an output layer that defines the size and type of output data. The experiment is described in detail in Table 3 and its description is in the Experimental Methodology and Result section.

#### 3.2.2. The Use of Transfer Learning

Another approach to work in Deep Learning is using a pre-trained Deep Neural Network. For the first approach, the advantage is its structure; a model of an already existing Deep Neural Network is used by applying a few simple changes. In the latter case, a limited data set is used and knowledge is transferred from this model to a new task. It is also said to transfer the learned characteristics of a pre-trained CNN to a new problem with a limited data set. Transfer learning involves forming a CNN with available labelled source data (called a source learner) and then extracting the inner layers that represent a generic representation of mid-level entities to a target CNN learner. An adaptation layer is added to the target CNN learner to correct for any different conditional distributions between the source and target domains. The experiments are performed on the object image classification, where the average precision is measured as a measure of performance. The first experiment was performed using the Pascal VOC 2007 dataset as the target and ImageNet 2012 as the source. The second experiment was performed using the Pascal VOC 2012 dataset as the target and ImageNet 2012 as the source. The tests have successfully demonstrated the ability to transfer information from one CNN learner to another [34].

The main advantage of transfer learning is that it does not need a lot of data to give a good accuracy (and this is true in most cases). Transfer learning has proven to be a solution to many real problems. Some of them are; for example [35], the transfer learning techniques were used to improve the global climate by classifying aerosol dust particles. In [36], and in using transfer learning tools, an approach has been proposed to be able to identify low-income areas in developing countries that are important for disaster relief efforts. In [37], transfer learning is used to improve disease prediction. In [38], transfer learning was used to improve the problem of facial recognition using the face image information of a source group to improve the learning of a classifier for a target group. In [39] transfer learning was applied to the field of biology. Therefore, the following concept was applied for the analysis of Nosema disease.

##### Nosema Recognition with Alexnet Classifier

Several architectures were examined, and AlexNet was one of them. AlexNet is one of the first pre-trained Neural Networks; it is trained using a large image dataset called ImageNet, which in turn contains more than millions of images and 22 thousand visual categories. AlexNet is trained on more than a million images and can classify images into 1000 object categories. This paper used the pre-trained weights of the AlexNet network, which contains 25 layers. Then, the network was fine-tuned for the classification problem by replacing the last three layers of AlexNet pre-trained model with a fully connected layer (layer number 23), a softmax layer (layer number 24) and a classification output layer (layer number 25). The new model was fine-tuned using 2000 input cell images for two classes: Nosema class and Non Nosema Class. Since AlexNet requires exactly 227 × 227 RGB input images, the images were automatically resized to this dimension during the data augmentation. The augmentation of the data helps prevent the network from overfitting and helps its better generalization capabilities. Furthermore, the data were split into two parts, one for training and the other for validation of results. Each experiment and its results are shown in Table 7, Section 4.

##### Nosema Recognition VGG-16 and VGG-19 Classifiers

VGG-16 and VGG-19 are another pre-trained neural network models. They are again pre-trained using ImageNet dataset. These two models were chosen because they learned a good representation of low-level characteristics such as space, edges, color, lighting, texture and shapes; and these characteristics are very useful for knowledge transfer and act as a feature extractor for new images. Since the images in this work belong to completely different categories from the source dataset, but the pre-trained model should still be able to extract relevant features from these images based on transfer learning principles. These pre-trained models—VGG-16 and VGG-19 were transferred again for classification of images of Nosema cells against images of other objects.

**Figure 6:**
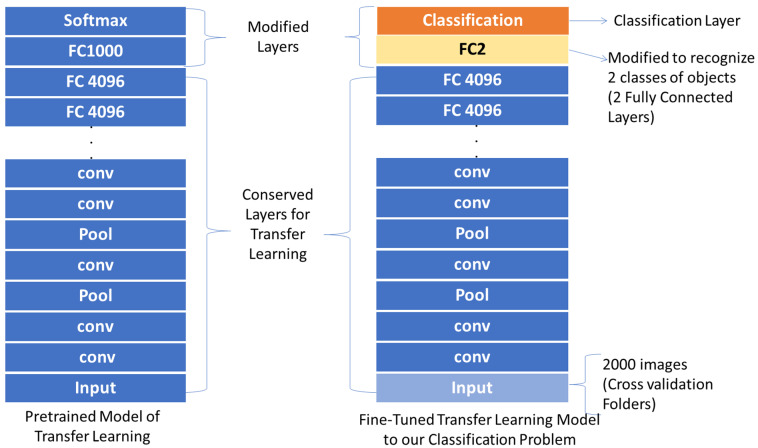
Modifying Transfer Learning Models for This Proposal.

VGG-16 pre-trained network contains 41 layers and VGG-19 contains 47 layers. The last three layers of VGG-16 and the number of layers 45 and 47 for VGG-19 were replaced with fully connected layers and trained with 1000 Nosema images and 1000 non-Nosema images. The network expects 224 × 224 RGB or grayscale input images, so the input images were resized. The dataset was split into learning and validation parts regarding different average of data division. Figure 6 shows the used model for modification of the pre-trained transfer learning models used in this paper.

## 4. Experimental Methodology and Results

For the statistical evaluation, the 10-fold cross-validation strategy was followed between 10% and 90%. Accuracy is used as a quality measure here. The experiments have been designed for machine learning approaches (SVM and ANN), transfer learning approaches (AlexNet, VGG-16 and VGG-19), and deep learning method with CNN.

The first experiment was done for ANN and SVM. For ANN, just a single hidden layer was used and only the number of neurons in the hidden layer was adjusted, using 15 or 19 neurons for the input layer and 1 neuron for the output layer (see Table 2).

The next experiment used the deep learning method, in particular deep CNN classifier. The architecture of CNN had 3 convolutional blocks, which have been stacked with 3 × 3 filters followed by a 2 × 2 subsampling layer (max_pooling). In this way, increasing the number of filters increases the depth of the network, and a kind of cone is formed with increasingly reduced but more relevant characteristics. It should be noted that in convolutional layers, padding is used to ensure that the height and width of the output feature maps match the inputs. Finally, each layer will use the ReLU activation function. Additionally, dropout layers have been added that implement regularization. The dropout technique is a simple technique that will randomly remove nodes from the network and has the effect of regularization as the remaining nodes must adapt to compensate for the slack of the removed nodes and a layer of batch normalization. Batch normalization (batch_normalization) is a technique designed to automatically standardize inputs to a layer in a deep learning neural network and has the effect of speeding up the process of training a neural network and, in some cases, improving the performance of the model. Once the above has been commented on, in Table 3, the architecture used for an 80 × 80 input image with three RGB channels is shown. The accuracy reached 92.50%.

Finally, the last experiment was for transfer learning approaches. AlexNet is known for its simplicity, but in the case of this experiment, it does not give an encouraging result. SGDM was the default and chosen optimizer for AlexNet. AlexNet does not require many options to work well, and the default training options were reserved. Sixty-four is the size of mini-bach and the initial learning rate was chosen as 0.001. The maximum number of epochs is fixed to 20; this chosen training options made the experiment faster (see Table 4). Table 5 describes the four cross-validation folders experiments and given accuracy by each one. As is shown in Table 5, the third experiments in which the data were split between 70% for training and 30% for test and validation, give the best accuracy (87.48%) by 6 epochs number.

Only the last three layers of VGG-16 and VGG19 were modified to make them fit the target domain. The fully connected layer (FC) in both models has been changed to a new FC layer with an output size of 2 according to the 2 classes, which were needed to classify. Adam was the chosen optimizer, given his good learning rate and the specific adaptive nature of the learning rate parameters. For Adam, the initial learning rate was chosen as 0.0004; a small valor is a good option to increase the training time. The size of the mini-batch was fixed at 10. The validation information of the model is that given in the test. Thus, a learning factor of 10 is defined. The maximum number of epochs was fixed to 25 but during the simulation process, the number was variable according to the experiments carried out, but it was initialized in the first experiment to 6. Finally, a validation frequency set to 3. The trained options of the experiment are listed in Table 4.

**Figure 7:**
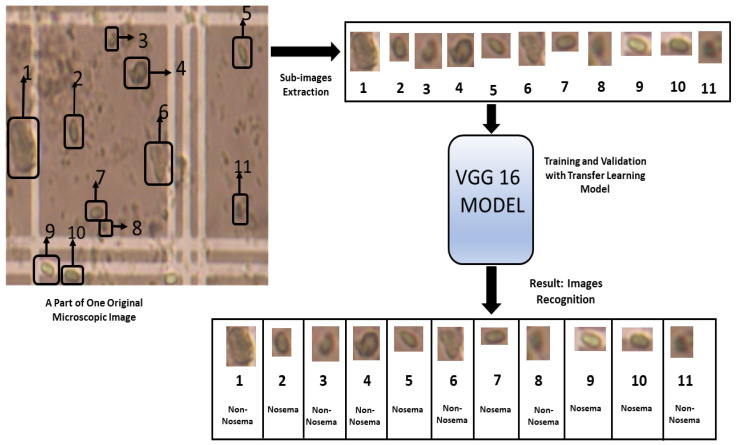
The steps followed for the recognition of Nosema cells using VGG 16 Model.

**Figure 8:**
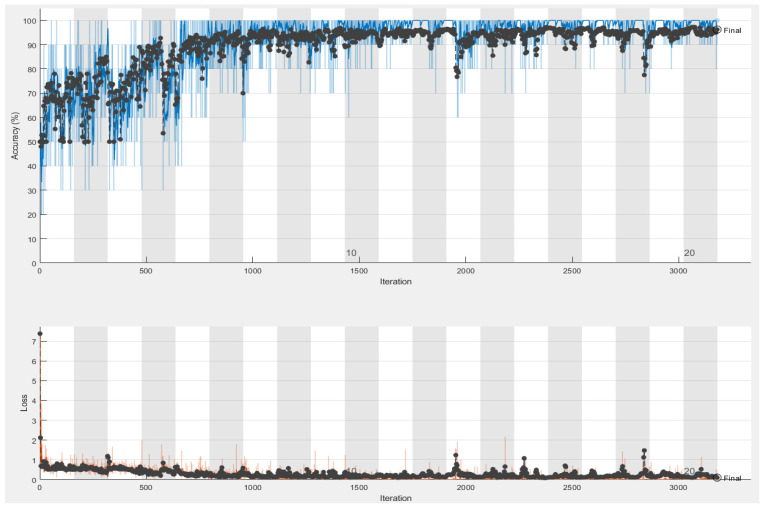
The Accuracy (blue curve) and loss (orange curve) results given by VGG-16 simulation: 96.25% of success accuracy with 20 epochs.

Detailed results for VGG-16 and VGG-19 neural networks are shown in Table 4, and while the best simulation accuracy is given by VGG-16, Figure 7 describes the followed steps using VGG16 to identify the Nosema and Figure 8 shows the best accuracy. Three experiments have been implemented, but only those that gave good results with a similar number of epochs for the two pre-trained networks have been described in Table 6. The data was split between training and validation, the experiments were conducted 30 times, following a 10-fold cross-validation process. The three last experiments gave the best accuracy; the first one took 70% of data for training and the 30% were for validation and the best accuracy was given by 6 epochs number. In the second experiment, 80% were placed for training, and the rest were for validation, the experiment was repeated several times with increasing the number of epochs and as Table 6 shows, the best accuracy given by VGG-16 is 96.25% with 20 epochs, and for VGG-19, the highest accuracy is 93.50% with 25 epochs, and in the third experiment presented in the result section, the data were divided between 90% for training and 10% for testing, and the results made an accuracy fall.

Table 7 summarizes the main results of the different experiments. The best result is reached using VGG-16 with accuracy of 96.25%, and the lowest accuracy is given by ANN (83.20%). Those results will be discussed in the next section.

## 5. Discussion

This section discusses in detail the behavior and features of each experiment and it discusses compromise between accuracy and the robustness of the proposed methods was included. Besides, a comparison vs. the most representative publication on this topic (see Table 8), with comparison vs. a previous work [14], authors increased the dataset from 185 to 2000 images and the extracted features number from 9 to 19, and those features for the Nosema cell are related to several aspects of the image cell: geometric shape, statistical characteristics, texture and color features given by GLCM. Two strategies were followed to recognize Nosema; while only one was followed (ANN) in [14]; the first strategy consists of the use of calculated characteristics by an ANN and an SVM and the second is based on sub-images extracted from treated microscopic images using an implemented CNN and the tools of transfer Learning. ANN used in [14] gave a success rate of 91.1% in Nosema recognition. SVM also was used in [13] to classify the two types of Nosema and other objects. The experiments reached relative and accurate values.

From Table 2, Table 3, Table 4, Table 5 and Table 6, it can be concluded that whether it is the largest dataset or the smallest dataset, the level of learning of the network with transfer learning models is obviously better than the traditional models, especially ANNs are examined in this study and SVM which brought near results. Furthermore, one notes a clear rate of convergence of the transfer model VGG-16 and VGG-19 at the level of the provided results. In addition, these transfer models are a bit faster than ANN and SVM, at least in this case. CNN has demonstrated its effectiveness in this problem of recognizing or classifying Nosema cells as a deep learning model. CNN was almost comparable to VGG-19. On the other hand, it should be said that the training options for the ANNs, as well as the transfer learning algorithms, make a difference in the results.

In front of AlexNet, the VGG-16, VGG-19 and CNN have proven their strong effectiveness in this work in the classification of patterns, cells and objects.

For the features extraction part, several different features from the sub-images were evaluated: geometric, statistic, texture and GLCM features extracted from the yellow channel. This experiment used a large database, the results given by the ANN as well as by the SVM good since it is the first time. The quality of the microscopic images used in this work did not always help to extract clear and sharp objects. By calculating the results with a different number of features (15 and 19), the importance of the data extracted by the GLCM in the resulting amelioration was approved.

## 6. Conclusions

In order to identify Nosema cells, this experiment examined two strategies of classification: the traditional ones and the deep learning classifiers. Different experiments were implemented for both strategies, despite the noisy quality of the microscopic images used. The best accuracy for the recognition or classification of Nosema is reached by VGG-16, 96.25%, which is compared to state of the art is the most accurate methodology in this area so far. 

The innovation of this proposal is to analyze and find the better option for this identification, checking different strategies to implement an automatic identification of Nosema cell, as was shown after experiments, and with good and robust accuracy. It was reached with VGG-16 architecture. 

After reviewing the state-of-the-art material, it can be concluded that only a few automatic approaches have been introduced so far. Because of this, we contribute with a variety of explored classification methods and their accuracies. In particular, we would emphasize the difference between shallow ANNs with handcrafted features and end-to-end learning using the deep learning approach using CNN together with several transfer learning architectures. 

## Figures and Tables

**Table 1:** Dataset of extracted sub-images.

Images	Number	Color	Type	Resolution
Nosema sub-images	1000	RGB	JPEG	229 × 161
Non-Nosema sub-images	1000	RGB	JPEG	450 × 257

**Table 2:** Results for experiments with ANN and SVM.

Number of Features	Classifier	Accuracy	Observation
15 Features	ANN	79.00%	For 1400 neurons in the hidden layer
	SVM	81.00%	Using kernel RBF
19 Features	ANN	83.20%	For 1400 neurons in the hidden layer
	SVM	83.50%	Using kernel RBF

**Table 3:** CNN architecture for an 80 × 80 input image.

Layer Type	Output Shape	Number of Parameters
conv2d (Conv2D)	(None, 80, 80, 32)	896
batch_normalization (BatchNo)	(None, 80, 80, 32)	128
conv2d_1 (Conv2D)	(None, 80, 80, 32)	9248
batch_normalization_1 (Batch)	(None, 80, 80, 32)	128
max_pooling2d (MaxPooling2D)	(None, 80, 80, 32)	0
dropout (Dropout)	(None, 80, 80, 32)	0
conv2d_2 (Conv2D)	(None, 80, 80, 64)	18,496
batch_normalization_2 (Batch)	(None, 40, 40, 64)	256
conv2d_3 (Conv2D)	(None, 40, 40, 64)	36,928
batch_normalization_3 (Batch)	(None, 40, 40, 64)	256
max_pooling2d_1 (MaxPooling2)	(None, 40, 40, 64)	0
dropout_1 (Dropout)	(None, 40, 40, 64)	0

**Table 4:** Experimental training parameters for AlexNet, VGG-16 and VGG-19.

Model	Parameters	Setting Values
AlexNet	Learning algorithm	Sgdm
	Initial Learning Rate	0.001
	Mini-batchsize	64
	Maximum epochs	0
VGG-16 and VGG-19	Learning algorithm	Adam
	Initial Learning rate	0.0004
	Mini-batch size	10
	Maximum epochs	25
	Validation Frequency	3
	Validation Information	Test-Images

**Table 5:** Cross-validation and simulation results for Alex-Net classifier.

Experiment (Trained Data, the Rest for Validation)	Accuracy	Epochs Number
0.5	84.58%	6
0.6	83.98%	6
0.7	86.98%	6
0.8	85.28%	6

**Table 6:** Cross-validation and simulation results for VGG-16 and VGG-19 classifiers.

Experiments	Epochs	Accuracy	
		VGG-16	VGG-19
0.7	6	76.29%	71.95%
08	6	92.50%	93.00%
	12	94.50%	82.00%
	20	96.25%	92.32%
	25	93.00%	93.50%
0.9	6	88.00%	77.00%

**Table 7:** A summary of best results given by the 6 used tools for Nosema classification.

ANN	SVM	CNN	AlexNet	VGG-16	VGG-19
83.20%	83.50%	92.5%	87.48%	96.25%	93.00%

**Table 8:** Results from other references for Nosema recognition.

Reference	Data Size	Method	Accuracy
[14]	185 images (1655 extracted features)	ANN	91.10%
This work	2000 images	ANN	83.20%
This work	2000 images	SVM	83.50%
This work	2000 images	CNN	92.50%
This work	2000 images	AlexNet	87.48%
This work	2000 images	VGG-16	96.25%
This work	2000 images	VGG-19	93.50%

## Data Availability

The data presented in this study are available on request from the corresponding author. The data are not publicly available due to starting state of the research.
